# Hydroxychloroquine Use in Lupus Patients during Pregnancy Is Associated with Longer Pregnancy Duration in Preterm Births

**DOI:** 10.1155/2017/2810202

**Published:** 2017-12-17

**Authors:** S. J. Kroese, M. J. H. de Hair, M. Limper, A. T. Lely, J. M. van Laar, R. H. W. M. Derksen, R. D. E. Fritsch-Stork

**Affiliations:** ^1^Department of Rheumatology and Clinical Immunology, University Medical Center Utrecht, Utrecht, Netherlands; ^2^Department of Gynecology and Obstetrics, University Medical Center Utrecht, Utrecht, Netherlands; ^3^Medizinische Abteilung Hanusch Krankenhaus and Ludwig Boltzmann Institut für Osteologie, Heinrich-Collin-Straße 30, 1140 Vienna, Austria; ^4^Sigmund Freud PrivatUniversität Wien, Vienna, Austria

## Abstract

**Objective:**

To investigate the effect of hydroxychloroquine (HCQ) in pregnant women with systemic lupus erythematosus (SLE).

**Methods:**

In SLE pregnancies of a single Dutch center (2000–2015), lupus activity and flares before and during pregnancy and postpartum were assessed using the SLE Disease Activity Index (SLEDAI)/SLEPDAI (SLEDAI adjusted for pregnancy). The association between HCQ use and pregnancy outcomes (early spontaneous abortion, fetal death, and preterm and term live birth) was analyzed using generalized estimating equations (GEE) accounting for the occurrence of multiple pregnancies per patient. Analyses were adjusted for antiphospholipid antibody (aPL) status.

**Results:**

110 pregnancies (63 mostly Caucasian patients) were included, of which, in 30, HCQ was used; overall occurrence of flares was low (non-HCQ group: 5 mild (6.4%) and 2 severe (2.6%); HCQ group: 2 mild (6.7%) and no severe flares). The HCQ group showed a trend towards lower dosage of prednisone (OR 0.2 (95% CI 0.0–1.4); *p* = 0.10). Pregnancy outcomes were comparable between groups. Among preterm live births, pregnancy duration was significantly longer in HCQ users (2.4 weeks (95% CI 1.0–3.8; *p* ≤ 0.001)).

**Conclusion:**

HCQ use was associated with longer pregnancy duration in the vulnerable preterm birth population, underscoring the beneficial effect of HCQ use during pregnancy.

## 1. Introduction

Pregnancy constitutes a challenge in patients with systemic lupus erythematosus (SLE). Apart from disease flares during pregnancy, SLE patients have an increased risk of intrauterine growth restriction (OR 2.6), (pre)eclampsia (OR 3.0), and preterm birth (OR 2.4) compared to the healthy population [1]. In SLE patients, the antimalarial drug hydroxychloroquine (HCQ) is not only used for the treatment of skin lesions and arthritis but also for a more general goal, namely, prevention of cardiovascular disease and flares. The use of HCQ during pregnancy has long been debated, but nowadays, consensus is reached that it is safe and it is frequently prescribed during pregnancy to diminish flares [2, 3]. However, reports on the detailed effects of HCQ on pregnancy outcomes are scarce and mostly focus on teratogenicity. Recently, a French retrospective study of 118 pregnancies in SLE patients who delivered after 22-week gestation or longer found less preterm birth and intrauterine growth restriction in women who used HCQ during pregnancy compared to those who did not use HCQ in the six months prior to or during pregnancy [4]. Our study is the first to investigate multiple pregnancies in SLE women in a tertiary center in order to provide insight into the effects of HCQ use on pregnancy outcomes in a (homogenous) Dutch SLE population.

## 2. Methods

Data of all pregnancies between 2000 and 2015 in women with SLE seen at the University Medical Center (UMC) Utrecht were retrieved from patient medical files, from an intern SLE registry, and from an in-house obstetric registry. SLE was classified according to the 1997 American College of Rheumatology (ACR) criteria [5]. Disease manifestations and duration at the start of pregnancy as well as treatment during pregnancy were recorded. Disease activity was assessed using the SELENA-SLEDAI score or SLEPDAI score where appropriate (SLEDAI adjusted for pregnancy) six months before and during pregnancy as well as six weeks postpartum [6, 7]. Flares were classified according to the SLE(P)DAI scores as mild/moderate or severe [6, 7]. Pregnancy outcomes were categorized according to pregnancy duration as early spontaneous abortion (<10 weeks of gestation), fetal death (>10 weeks of gestation), preterm live birth (PTLB; live birth < 37 weeks), and term live birth (TLB; live birth ≥ 37 weeks). Terminated pregnancies due to social reasons were not included in this study. Preeclampsia was defined as hypertension (>140 mmHg systolic or >90 mmHg diastolic with previous normal tension) combined with proteinuria (>0.3 g/24 h) [8]. HELLP (hemolysis, elevated liver enzymes, and low platelets) syndrome was defined following the Tennessee criteria as high LDH (>600 U/l) combined with low platelets (<100 × 10^9^/l) and elevated liver enzymes (ASAT > 70) [9]. Incomplete HELLP (iHELLP) fulfilled one or two of the required three items of the HELLP syndrome. Small for gestational age (SGA) was defined as birth weight below the 10th percentile (<p10). The Ethics Committee at the UMC Utrecht provided approval for the SLE registry, and all patients gave written informed consent.

Data were described using mean and standard deviation (SD) or median and interquartile range (IQR) where appropriate. As several of the patients had had multiple pregnancies, the association between HCQ use and pregnancy outcomes as well as pregnancy duration was analyzed using generalized estimating equations (GEE), correcting for patient dependency of observations, using an exchangeable correlation structure. GEE corrects for within-subject correlations in repeated measurements by the use of a correlation structure for the repeated measurements and corrects for standard errors of the regression coefficient [10, 11].

All analyses were adjusted for antiphospholipid antibody (aPL) status (based on the presence or absence of lupus anticoagulant and/or IgG or IgM class anticardiolipin antibodies and/or IgG or IgM anti-*β*2-glycoprotein antibodies) except for early spontaneous abortion, for which aPL occurrence was too infrequent for analysis. For the outcomes pregnancy duration, (pre)eclampsia, and (i)HELLP, only pregnancies with duration > 10 weeks of gestation were included (*n* = 89). A two-sided *p* value < 0.05 was considered statistically significant for all analyses. Statistical analysis was performed using IBM SPSS Statistics version 21.

## 3. Results

**Figure 1:**
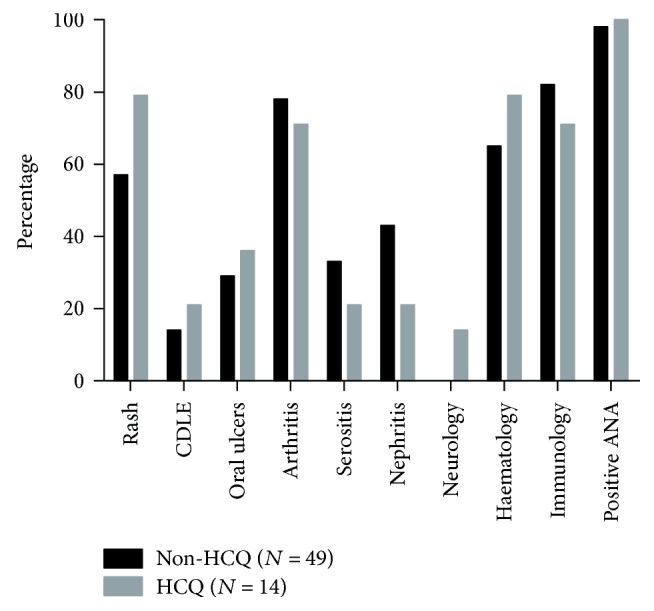
Percentage of patients fulfilling each ACR criterion cumulatively from the start of every first registered pregnancy. Results are separately shown for patients who used HCQ or did not use HCQ during the first registered pregnancy. CDLE: chronic discoid lupus erythematosus; ANA: antinuclear antibodies.

Sixty-three SLE patients were included. Their baseline characteristics are presented in Table 1. The percentage of patients fulfilling each ACR criterion cumulatively from the start of each first recorded pregnancy is shown in Figure 1. Neurology was the only ACR criterion that was found significantly more often in the HCQ group (*p* < 0.05); however, absolute numbers are small (*N* = 2 (HCQ) versus *N* = 0 (non-HCQ)).

Of the total group of 63 SLE patients, 110 pregnancies were included. Of the 63 patients, 14 used HCQ in 30 pregnancies. The dose of HCQ used in these pregnancies was 200 mg/day (16 pregnancies) or 400 mg/day (14 pregnancies). Ninety-six of 110 pregnancies (87%) were planned and beforehand medically approved by a rheumatologist or gynecologist at counselling consultations.

Pregnancy outcomes according to HCQ use are shown in Table 2. Hypertensive disorder of pregnancy occurred in 17% of pregnancies, and over 60% of pregnancies ended in full-term birth. Fourteen of eighteen preterm births were iatrogenically induced preterm deliveries due to either maternal (*n* = 7), fetal (*n* = 6), or combined (*n* = 1) indications. Indications included a.o. solutio placentae, severe preeclampsia, and placental insufficiency. There were no statistically significant differences in the occurrence of preterm birth between both groups; however, a substantial, although not significantly proportional, part of patients showed lower occurrence within the HCQ group (6.7% with HCQ use versus 20.0% without HCQ, OR 0.5 (95% CI 0.1–2.4); *p* = 0.37). Among the PTLB, pregnancy duration was significantly longer (2.4 weeks (95% CI 1.0–3.8), *p* ≤ 0.001) for pregnancies during which HCQ was used than for those during which no HCQ was used. A prolongation of pregnancy was not observed in pregnancies which ended in term birth. The association between HCQ use and pregnancy duration in PTLB was not confounded by the use of prednisone or azathioprine. However, in HCQ users who smoked, a known risk factor for preterm birth, pregnancy duration was only 1.9 weeks longer (95% CI 0.6–3.2; *p* = 0.01) instead of 2.4 weeks. The rate of SGA children did not statistically significantly differ between the non-HCQ and HCQ groups (12.5 versus 16.7%, OR 2.2 (95% CI 0.6–7.5); *p* = 0.22). One case of neonatal lupus occurred: a congenital heart block in the non-HCQ group. Congenital anomalies included one case of, respectively, hip dysplasia, Loeys-Dietz syndrome, and anal atresia, all in the non-HCQ group.

The median SLE(P)DAI score was 2 (IQR 0–4) six months before conception, for each trimester and for the 6-week postpartum period, irrespective of HCQ use. Six months before conception, there were no severe flares and two mild flares in both groups (6.7% (HCQ) versus 2.5% (non-HCQ)). Mild flare occurrence was 0/2/3/3 in the non-HCQ group and 1/0/1/3 in the HCQ group per trimester and postpartum. Two severe flares occurred, both in the non-HCQ group, in the first and second trimester. Flares were usually characterized by arthritis (40%), low complement levels (27%), proteinuria (27%), and/or a rise in anti-dsDNA (20%).

In 57% of pregnancies, prednisone was used (Table 2). HCQ use was not associated with prednisone use (53.8% (non-HCQ) versus 60.0% (HCQ), OR 0.9 (95% CI 0.7–1.2); *p* = 0.35). However, in the pregnancies with prednisone therapy, there were distinctively more patients with a higher dosage of prednisone (>7.5 mg) in the non-HCQ group compared to the HCQ group, although this did not reach statistical significance (17.5% (non-HCQ) versus 73.3% (HCQ), OR 0.2 (95% CI 0.0–1.4); *p* = 0.13). The use of azathioprine did not differ between the non-HCQ and HCQ pregnancies.

## 4. Discussion

In this study, we investigated the association between HCQ use and disease activity as well as pregnancy outcomes in the SLE population followed at a single center. Our most important finding was that HCQ was significantly associated with longer pregnancy duration in the subgroup of women with preterm delivery. To our knowledge, this has not been reported in SLE pregnancies yet. Several recent studies have shown that neonates of HCQ-using pregnant SLE women reach a higher gestational age at birth [3, 4, 12]. Although this is in line with our findings, none of these described the effect of HCQ on preterm births.

Since fetal cerebral and respiratory developments are known to improve with the length of pregnancy, especially in preterm births, the gain in pregnancy duration of more than a week in this subgroup is of great clinical relevance [13, 14]. A possible explanation for the longer pregnancy duration in HCQ users is the recently described positive effects of antimalarials on endothelial dysfunction, which might preserve placental function and thus pregnancy [15].

Although we did not find a significantly lower occurrence of preterm birth within the HCQ group as described by Leroux et al. [4] (15.8% versus 44.2% (*p* = 0.006)), a trend towards less preterm births within the HCQ group was observed in our study [12].

We did not find a significantly higher rate of SGA in the non-HCQ group compared to the HCQ group as described in other studies [4, 16]. The higher rate of SGA in non-HCQ users in the French cohort compared to our cohort (26% versus 6%) could be explained by the higher occurrence of SLE flares in the former. Previous studies described a trend towards lower SLE disease activity in pregnancies with HCQ use [2, 17]. In our study, disease activity scores and frequency of flares during pregnancy were not significantly different between both groups. This might be due to the fact that our population comprised predominantly Caucasian women with quiescent lupus at the start of pregnancy. The latter is a consequence of the policy in our center to aim at planning parenthood and extensive prepregnancy counselling. In our patient group, 87 percent of pregnancies were considered safe by the consulting physician in prepregnancy counselling. Quiescent disease for at least six months prior to pregnancy is known to reduce flare rates during pregnancy [18].

In our study, women using HCQ showed a trend towards lower prednisone dose throughout their pregnancies, albeit not statistically significant. In a prospective study of 357 pregnancies, a similar trend towards lower prednisone dose during pregnancy was noted within the group of patients using HCQ [2].

The limitation of our study is its retrospective and observational character. Due to the low number of adverse outcomes, our findings might underestimate the effect of HCQ and might be influenced by a type II error. Although the use of HCQ in SLE pregnancies is nowadays encouraged, a recent prospective study analyzing (adverse) outcomes and predictors thereof in pregnancies of SLE patients shows that only 64.7% used HCQ during pregnancy [19]. No data are provided on the effect of this treatment on prolongation of pregnancy duration in preterm deliveries. To confirm that treatment with HCQ in pregnant patients with SLE is beneficial for pregnancy outcome, a large, prospective, double-blinded randomized clinical trial is needed. However, given the well-established role for HCQ as one of the cornerstones of SLE treatment and the results from a recent meta-analysis that confirmed that HCQ can safely be used during pregnancy [16], such a trial does not seem ethical. Our findings provide further grounds for the continuation or even start of HCQ in SLE patients who want to become pregnant.

## Figures and Tables

**Table 1:** Patient characteristics of SLE patients.

	Total (*N* = 63)	Non-HCQ (*N* = 49)	HCQ (*N* = 14)
Race (*n* (%))			
Caucasian	56 (89)	45 (92)	11 (79)
Black	1 (2)	1 (2)	0 (0)
Asian	6 (10)	3 (6)	3 (21)
Age at the start of the first registered pregnancy (mean (SD))	31.0 (4.1)	30.6 (4.0)	32.5 (4.3)
Disease duration at the start of the first registered pregnancy	6.0 (3.0–10.0)	6 (3.5–9.5)	4.5 (3–10.8)
Number of registered pregnancies per patient	1 (1-2)	1 (1-2)	2 (1-2)
Antiphospholipid antibodies present	7 (11)	6 (13)	1 (7)
LAC (*n* (%))^∗^			
Negative	58 (92)	44 (90)^∗^	14 (100)
Positive	4 (7)	4 (9)	0 (0)
aCL-IgG (*n* (%))			
Negative	59 (94)^∗^	45 (92)^∗^	14 (100)
Positive	3 (54)	3 (6)	0 (0)
aCL-IgM (*n* (%))			
Negative	61 (97)^∗^	48 (98)^∗^	13 (93)
Positive	1 (2)	0 (0)	1 (7)

SD: standard deviation; IQR: interquartile range. Age and disease duration in years. Data depicted as median (IQR) unless otherwise indicated. ^∗^Unknown: *n* = 1. Anti-beta2 glycoprotein IgG/IgM was tested in 31 patients in whom it was all negative.

**Table 2:** Maternal and fetal pregnancy outcome according to HCQ treatment.

	Total (*N* = 110)	Non-HCQ (*N* = 80)	HCQ (*N* = 30)	OR (95% CI)^$$^; *p* value
*Maternal outcome*				
Preeclampsia^∗^	13 (11.8)	9 (11.3)	2 (6.7)	1.0 (1.0-1.0); 0.57
Eclampsia^∗^	0 (0)	0 (0)	0 (0)	—
(i)HELLP^∗^	6 (5.5)	5 (6.3)	1 (3.3)	1.3 (0.1–17.9); 0.84
Prednisone use^†^	63 (57.3)	43 (53.8)	18 (60.0)	0.9 (0.7–1.2); 0.35
Prednisone < 7.5 mg within prednisone users	36 (32.7)	14 (17.5)	22 (73.3)	0.2 (0.0–1.4); 0.10
*Fetal outcome*				
Early spontaneous abortion (<10 weeks of gestation)	19 (17.3)	10 (12.5)	9 (30.0)^▲^	1.5 (0.3–9.0); 0.66
Fetal death^‡^ (>10 weeks of gestation)	3 (2.7)	2 (2.5)	1 (3.3)	—
Preterm live birth	18 (16.4)	16 (20.0)	2 (6.7)	0.5 (0.1–2.4); 0.37
Of which <34 weeks	5 (4.5)	5 (6.3)	0 (0)	—
Term live birth	70 (63.6)	52 (65.0)	18 (60.0)	0.9 (0.3–2.7); 0.90
Small for gestational age	15 (13.6)	10 (12.5)	5 (16.7)	2.2 (0.6–7.5); 0.22
				*β* (95% CI)^$$^; *p* value
Duration of pregnancy^∗^ (median, IQR)	38.9 (37.1–40.0)	38.9 (36.4–40.1)	38.7 (37.7–39.4)	−1 (−3.8 to 1.8); 0.48
Duration of pregnancy in preterm live births^#^ (median, IQR)	35.1 (31.5–36.3)	34.9 (30.9–35.4)	36.8 (36.7-...)	2.4 (1.0–3.8); 0.001

Data depicted as numbers (%) unless otherwise indicated. HCQ: hydroxychloroquine; IQR: interquartile range; HELLP: (incomplete) hemolysis, elevated liver enzymes, and low platelet syndrome. ^$$^Dependent variable: pregnancy outcome/prednisone use/duration of pregnancy. Predictor variable: HCQ use (ref = non-HCQ). Adjusted for antiphospholipid status, except for early spontaneous abortion. ^∗^Pregnancies ending < 10 weeks of gestation were excluded (*N* = 89/68/21). ^†^Prednisone dose was increased in 4.6% of pregnancies. ^▲^Of which, 5 occurred within one woman. ^‡^Two were due to elective termination, one because of trisomy 21 with Fallot's tetralogy, and one because of infaust prognosis with severe preeclampsia, both occurring within the non-HCQ group. ^#^(*N* = 18/16/2) duration of pregnancy in weeks.
