# Corrigendum to “Hydroxychloroquine Use in Lupus Patients during Pregnancy Is Associated with Longer Pregnancy Duration in Preterm Births”

**DOI:** 10.1155/2022/9783521

**Published:** 2022-05-09

**Authors:** S. J. Kroese, M. J. H. de Hair, M. Limper, A. T. Lely, J. M. van Laar, R. H. W. M. Derksen, R. D. E. Fritsch-Stork

**Affiliations:** ^1^Department of Rheumatology and Clinical Immunology, University Medical Center Utrecht, Utrecht, Netherlands; ^2^Department of Gynecology and Obstetrics, University Medical Center Utrecht, Utrecht, Netherlands; ^3^Medizinische Abteilung Hanusch Krankenhaus and Ludwig Boltzmann Institut für Osteologie, Heinrich-Collin-Straße 30, 1140 Vienna, Austria; ^4^Sigmund Freud PrivatUniversität Wien, Vienna, Austria

In the article titled “Hydroxychloroquine Use in Lupus Patients during Pregnancy Is Associated with Longer Pregnancy Duration in Preterm Births” [1], the authors discovered some discrepancies in the data which were used in the article. Thus, the authors repeated the analyses with the correct data. There was a slightly lower incidence of HELLP (4 in total of which 3 in the non-HCQ group) instead of 6 in total (of which 5 in the non-HCQ group), but statistical significance was similar. Other outcomes were unchanged.

Corrected Table 2 is shown below.

Additionally, the email address of the corresponding author should be changed to “s.kroese@hagaziekenhuis.nl.”

The authors confirm that this does not affect the results and conclusions of the article, and the editorial board agrees to the publication of a corrigendum.

## Figures and Tables

**Table 2:** Maternal and fetal pregnancy outcome according to HCQ treatment.

	Total (*N* = 110)	Non-HCQ (*N* = 80)	HCQ (*N* = 30)	OR (95% CI) ^$$^; *p* value
*Maternal outcome*				
Preeclampsia^∗^	11 (10.9)	9 (11.3)	2 (6.7)	1.0 (1.0-1.0); 0.997
Eclampsia^∗^	0 (0)	0 (0)	0 (0)	—
(i) HELLP^∗^	4 (3.6)	3 (3.8)	1 (3.3)	0.93
Prednisone use^†^	63 (57.3)	45 (56.3)	18 (60.0)	0.9 (0.7-1.2); 0.35
Prednisone < 7.5 mg within prednisone users	36 (32.7)	14 (17.5)	22 (73.3)	0.2 (0.0-1.4); 0.10
*Fetal outcome*				
Early spontaneous abortion (<10 weeks of gestation)	19 (17.3)	10 (12.5)	9 (30.0)^▲^	1.5 (0.3-9.0); 0.66
Fetal death^‡^ (>10 weeks of gestation)	3 (2.7)	2 (2.5)	1 (3.3)	-
Preterm live birth	18 (16.4)	16 (20.0)	2 (6.7)	0.5 (0.1-2.4); 0.37
Of which <34 weeks	5 (4.5)	5 (6.3)	0 (0)	-
Term live birth	70 (63.6)	52 (65.0)	18 (60.0)	0.9 (0.3-2.7); 0.90
Small for gestational age	15 (13.6)	10 (12.5)	5 (16.7)	2.2 (0.6-7.5); 0.22
				*β* (95% CI)^$$^; *p* value
Duration of pregnancy^∗^ (median, IQR)	38.9 (37.1-40.0)	38.9 (36.4-40.1)	38.7 (37.7-39.4)	-1 (-3.8-1.8); 0.48
Duration of pregnancy in preterm live births^#^ (median, IQR)	35.1 (31.5-36.3)	34.9 (30.9-35.4)	36.8 (36.7-..)	2.4 (1.0-3.8); 0.001

Data depicted as numbers (%) unless otherwise indicated. HCQ: hydroxychloroquine; IQR: interquartile range; HELLP: (incomplete) hemolysis, elevated liver enzymes, and low platelet syndrome. ^$$^Dependent variable: pregnancy outcome/prednisone use/duration of pregnancy. Predictor variable: HCQ use (ref = non-HCQ). Adjusted for antiphospholipid status, except for early spontaneous abortion. ^∗^Pregnancies ending <10 weeks of gestation were excluded [*N* = 89/68/21]. ^†^Prednisone dose was increased in 4.6% of pregnancies. ^▲^Of which 5 occurred within one woman. ^‡^Two were due to elective termination one because of trisomy 21 with Fallot's tetralogy and one because of infaust prognosis with severe preeclampsia, both occurred within the non-HCQ group. ^#^[*N* = 18/16/2], duration of pregnancy in weeks.
